# The Co-Culture of Staphylococcal Biofilm and Fibroblast Cell Line: The Correlation of Biological Phenomena with Metabolic NMR^1^ Footprint

**DOI:** 10.3390/ijms22115826

**Published:** 2021-05-29

**Authors:** Joanna Czajkowska, Adam Junka, Jakub Hoppe, Monika Toporkiewicz, Andrzej Pawlak, Paweł Migdał, Monika Oleksy-Wawrzyniak, Karol Fijałkowski, Marcin Śmiglak, Agata Markowska-Szczupak

**Affiliations:** 1 Laboratory of Microbiology, Łukasiewicz Research Network–PORT Polish Center for Technology Development, 54-066 Wrocław, Poland; joanna.czajkowska@port.lukasiewicz.gov.pl; 2 Department of Chemical and Process Engineering, West Pomeranian University of Technology, 71-065 Szczecin, Poland; monika.oleksy-wawrzyniak@umed.wroc.pl (M.O.-W.); agata.markowska@zut.edu.pl (A.M.-S.); 3 Department of Pharmaceutical Microbiology and Parasitology, Faculty of Pharmacy Wrocław Medical University, 50-556 Wrocław, Poland; 4 Poznan Science and Technology Park (PPNT), Rubiez 5, 61-612 Poznań, Poland; Jakub.Hoppe@ppnt.poznan.pl (J.H.); Marcin.Smiglak@ppnt.poznan.pl (M.Ś.); 5 Bioimaging Laboratory, Łukasiewicz Research Network—PORT Polish Center for Technology Development, 54-066 Wrocław, Poland; monika.toporkiewicz@port.lukasiewicz.gov.pl; 6 Department of Nervous System Diseases, Kazimierza Bartla 5, 50-996 Wrocław, Poland; andrzej.pawlak@umed.wroc.pl; 7 Department of Environment Hygiene and Animal Welfare, Wroclaw University of Environmental and Life Sciences, 51-630 Wroclaw, Poland; pawel.migdal@upwr.pl; 8 Department of Microbiology and Biotechnology, Faculty of Biotechnology and Animal Husbandry, West Pomeranian University of Technology, 70-311 Szczecin, Poland; karol.fijalkowski@zut.edu.pl

**Keywords:** *S. aureus*, fibroblasts, co-culture, infection, metabolic profiles, NMR

## Abstract

*Staphylococcus aureus* is one of the most prevalent pathogens associated with several types of biofilm-based infections, including infections of chronic wounds. Mature staphylococcal biofilm is extremely hard to eradicate from a wound and displays a high tendency to induce recurring infections. Therefore, in the present study, we aimed to investigate in vitro the interaction between *S. aureus* biofilm and fibroblast cells searching for metabolites that could be considered as potential biomarkers of critical colonization and infection. Utilizing advanced microscopy and microbiological methods to examine biofilm formation and the staphylococcal infection process, we were able to distinguish 4 phases of biofilm development. The analysis of staphylococcal biofilm influence on the viability of fibroblasts allowed us to pinpoint the moment of critical colonization—12 h post contamination. Based on the obtained model we performed a metabolomics analysis by ^1^H NMR spectroscopy to provide new insights into the pathophysiology of infection. We identified a set of metabolites related to the switch to anaerobic metabolism that was characteristic for staphylococcal biofilm co-cultured with fibroblast cells. The data presented in this study may be thus considered a noteworthy but preliminary step in the direction of developing a new, NMR-based tool for rapid diagnosing of infection in a chronic wound.

## 1. Introduction

The treatment of infected chronic wounds remains a global challenge for health care systems and consumes about 1–3% of European Union public health budgets [1]. Regardless of the type (diabetic foot, burns, venous leg ulcers, pressure ulcers), if managed inadequately, chronic wounds cause a dramatic deterioration of patients’ life quality [2]. Moreover, the complications caused by microbial biofilm settled within chronic wounds pose a risk of amputation, development of a systemic infection and, as a consequence, may lead to a deterioration of a patient’s health or even their death [3].

Devoid of the skin’s protective barrier and repeatedly flooded with nutrient-rich exudate, chronic wounds are attractive niches for microbial colonization and formation of biofilm. Thanks to the production of a changeable, extracellular matrix and owing to a metabolic differentiation of the cells within it, this dense community of microbes displays an extremally high tolerance/resistance to antimicrobials and the immune system [4]. The results of recent studies indicate that biofilm is the dominant form of microbial existence in wounds [5].

It is widely accepted that multiplication of microorganisms does not disturb the wound healing process until the specific point referred to as critical colonization. If this crucial stage occurs, medical intervention (biofilm removal) is required. If it is not performed, the process of microbial multiplication accelerates together with tissue damage and, to a various extent, with inflammatory response [6]. This phenomenon is referred to as infection. In the case of infected chronic wounds, the majority of wound treatment guidelines recommend surgical removal (debridement) of disease-altered tissue followed by application of a modern dressing and aggressive treatment with antiseptics [7]. Nevertheless, the above-mentioned harsh procedures often prove ineffective, due to biofilm high adaptivity and its tolerance to the counter-measures applied.

If an adequate sanitary regime is preserved in nosocomial conditions, the majority of wound-colonizing pathogens come from the patient’s own microbiome (skin and gut, especially). The results of recent research indicate that chronic wounds may be colonized by microbial consortia formed by an abundance of species [8]. However, due to a vast arsenal of offensive and defensive virulence factors, only some of them pose a major concern with regard to treatment [9].

Undoubtedly, *Staphylococcus aureus* is one of such opportunistic pathogens. This Gram-positive coccus is considered a part of normal skin microbiome [10]. Although *S. aureus* is equipped with numerous toxins and tissue-degrading enzymes, it seems to be able to downregulate their expression (most people are considered asymptomatic carriers of this pathogen, especially in nostrils) [11]. However, a compromission of the human immune system and transgression of *S. aureus* to normally sterile niches of the organism (such as the blood system or the subcutaneous tissue forming the wound bed) activate *S. aureus* to release its virulence factors and to damage the eukaryotic cells [12]. Additionally, the methicillin resistance (MR) pattern which is more and more common among staphylococci and which leads to resistance against basically all β-lactam antibiotics has become a global threat. Therefore, rapid detection of *Staphylococcus* biofilm presence within the wound (and its removal with the use of non-antibiotic measures) is a matter of paramount importance from the point of view of effective antimicrobial treatment. It should be stressed that the existing methods of staphylococcal biofilm detection within the wound have certain disadvantages with regard to their accuracy, cost or sensitivity. The provision of a new diagnostic tool is a must if chronic wounds, aggravated by staphylococcal biofilm-based infections, are to be treated properly. Searching for an appropriate sample material for the above-mentioned purpose, we turned our attention to the wound exudate. This fluid is emitted by an organism in a process known as exuding or exudation. In wounds, exudate leaks out of the blood vessels into the wound bed [13]. Noteworthy, this fluid is composed not only of serum, fibrin, and leukocytes (from blood) but it also contains a set of metabolites—small molecules secreted from wound cells (of microbial or eukaryotic origin). We hypothesized that such type of sample could provide us with data concerning a potential presence of biofilm within the wound. To check this possibility, we applied advanced NMR ^1^H spectroscopy, frequently applied for various types of -omics study. NMR spectroscopy allows for the analysis of intra- (metabolic fingerprint) and extra-cellular (metabolic footprint) metabolites of low molecular weight, providing valuable insight into biochemical processes taking place within and outside of living cells. The results of clinical study we performed previously [14] (analysis of metabolic composition of exudate collected from infected leg ulcers) provided us abundance of data and clearly indicated that high proportion of identified metabolites are of common origin, i.e., they are secreted by both cells of patients and microorganisms themselves. This phenomenon significantly impeded choice of appropriate biomarker of ongoing infection. Therefore, in the present line of investigation, we explicitly undertook the reductionistic approach, opposite to the previous one. We co-cultured eukaryotic fibroblast line with *S. aureus* cells to obtain the in vitro model of chronic wound colonization/infection and we analyzed footprint metabolites secreted in specific time-points searching for potential biomarkers of staphylococcal biofilm-based infection. The rationale behind this agenda was to reduce (being fully aware of possible disadvantages of such approach) all phenomena occurring within wound in vivo to the core interactions occurring in in vitro setting between wound-healing cells and invading cells of *S. aureus*.

## 2. Results

**Figure 1:**
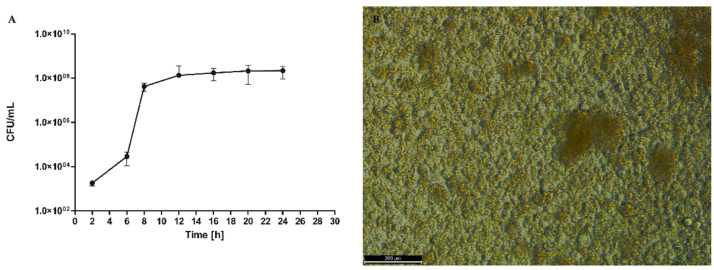
(**A**). *S. aureus* ATCC *6538* biofilm growth curve during 24 h of culturing. (**B**). Picture of *S. aureus* mature biofilm after 24 h post-inoculation taken with inverted microscope Leica DMIL LED, scale bar—200 µm, magnification 10×, Cfu—colony forming units.

In the first step of the investigation, we have conducted a series of experiments to analyze the biofilm formation process of *S. aureus ATCC 6538 strain*. We performed quantitative culturing as a preliminary (but robust) technique. (Figure 1) The results indicated that cultured alone, this particular strain multiplied exponentially to the eighth hour after inoculation and then reached a growth plateau lasting to the 24th h post-inoculation.

**Figure 2:**
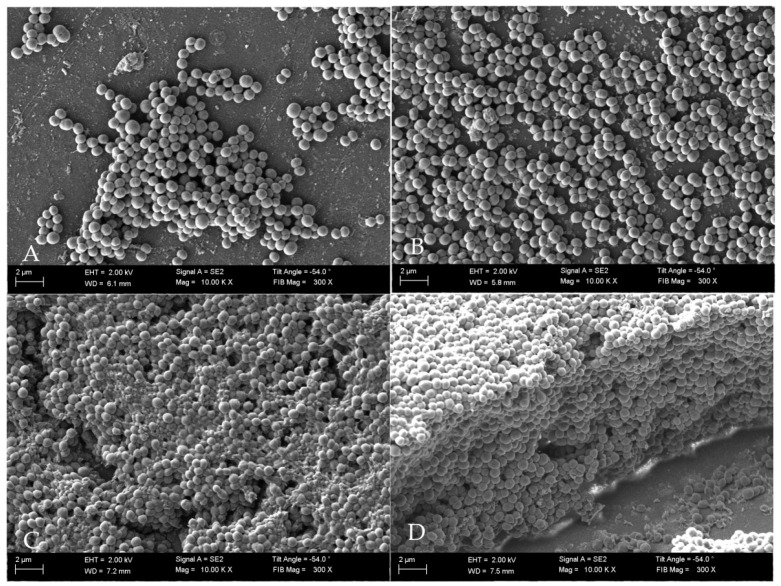
Images showing biofilm maturation process. (**A**) Initial attachment phase, (**B**) cell aggregation and accumulation of biomass, (**C**) maturing biofilm; (**D**) mature biofilm. SEM Auriga 60 microscope, magn. 10,000×.

To correlate the quantitative results of cell number with individual stages of staphylococcal biofilm formation we applied Scaning Electron Microscopy. The obtained data allowed us to indicate the specific phases of biofilm development—adhesion (Figure 2A) occurred during the first hours post inoculation, cell aggregation and accumulation of biomass (Figure 2B) (observed between the second and sixth hours of inoculation), and maturation (Figure 2C), lasting to ca.12th h from inoculation. Mature biofilm was observed from the 12th h to the end-point of this analysis (24 h) (Figure 2D). Noteworthy, in the 20th h of biofilm culturing, relatively high standard deviations of the average cell number occurred.

Having identified the specific stages of staphylococcal biofilm formation we analyzed the growth ratio of fibroblasts (wound healing cells). Our aim was to establish the moment/concentration, when these cells covered the surface with high cofluency resembling the wound bed in vivo.

**Figure 3:**
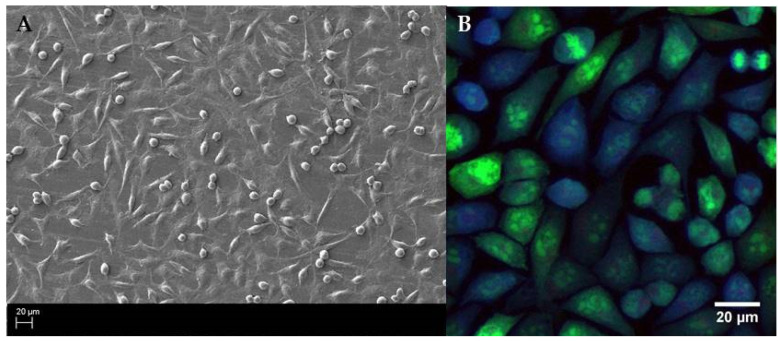
Image of a dense monolayer of fibroblast cells, after 16 h of culture imaged by (**A**) electron scanning microscopy, magnification 500×. And (**B**) confocal microscopy using CellTrace Violet^®^ and Syto 9^®^ (magnification 40×, scale bar 20 µm). It should be noted that the SEM picture allowed to confirm cofluence of fibroblasts and their proper (elongated) morphological shape, while confocal microscopy, coupled with the application of specific dyes, confirmed the fibroblasts’ viability.

After testing various initial concentrations, we have chosen the inoculum of 2 5 × 10^5^ fibroblast cell/1 mL. The application of such quantity of fibroblasts allowed us to obtain a basically cofluent (covered completely with live cells) surface after 16 h post inoculation as was confirmed by SEM (density of cells) and confocal microscopy (density and viability of cells) (Figure 3).

**Figure 4:**
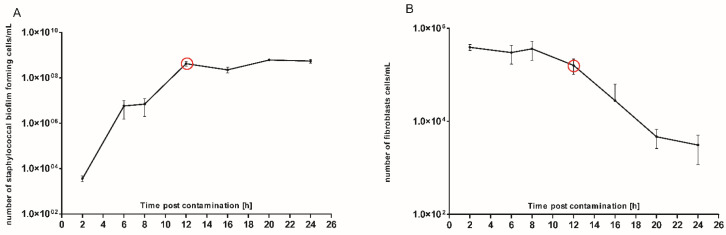
Co-culture of biofilm-forming *S. aureus* with L929 cells. (**A**) Growth dynamics of bacterial biofilm; (**B**) alterations of fibroblast cell number. Red circles indicate the moment [12th h] of reaching a growth plateau by staphylococcal biofilm and co-current drop in fibroblast cell number.

The analysis of data presented in Figure 1, Figure 2 and Figure 3 allowed us to establish proper conditions for *Staphylococcus* and fibroblast co-culture setting. We introduced 1.5 × 10^3^ CFU/mL of *S. aureus* planktonic (non-biofilm, free-swimming) cells to a cofluent fibroblast monolayer. The co-culture process was carried out for 24 h, during which the number of staphylococcal cells/fibroblast viability was recorded in the time-points indicated in Figure 4. The primary goal of this analysis was to pinpoint the specific moment during staphylococcal colonization at which the number of fibroblast cells starts to significantly decrease. Based on the data presented in Figure 4 we assumed that this specific time-point occurred approx. in the 12th h of colonization.

**Figure 5:**
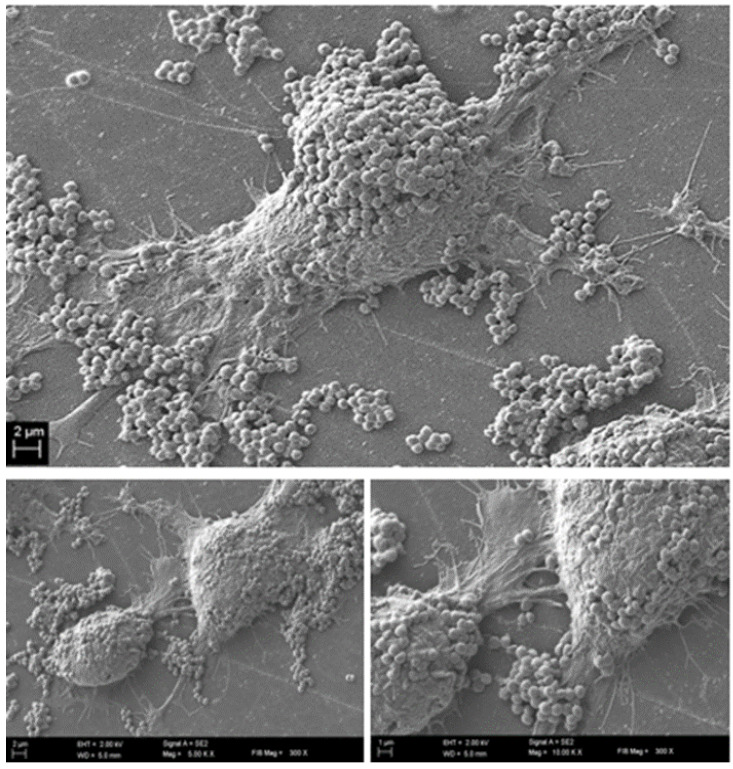
Co-culture of fibroblast ad staphylococci 12 h post contamination—reflecting critical colonization stage of infection. Magnification 5000, and 10,000×, respectively.

To confirm this assumption and to get a comprehensive insight into the phenomena occurring at this specific time point, we performed further analyses (electron scanning microscopy and confocal microscopy). The results of SEM visualization proved that after twelve hours post contamination, the number of fibroblast cells within the field of vision started to drop significantly. Moreover, the survived fibroblasts were covered with numerous *S. aureus* aggregates, tightly adhering to eukaryotic cells surface (Figure 5).

**Figure 6:**
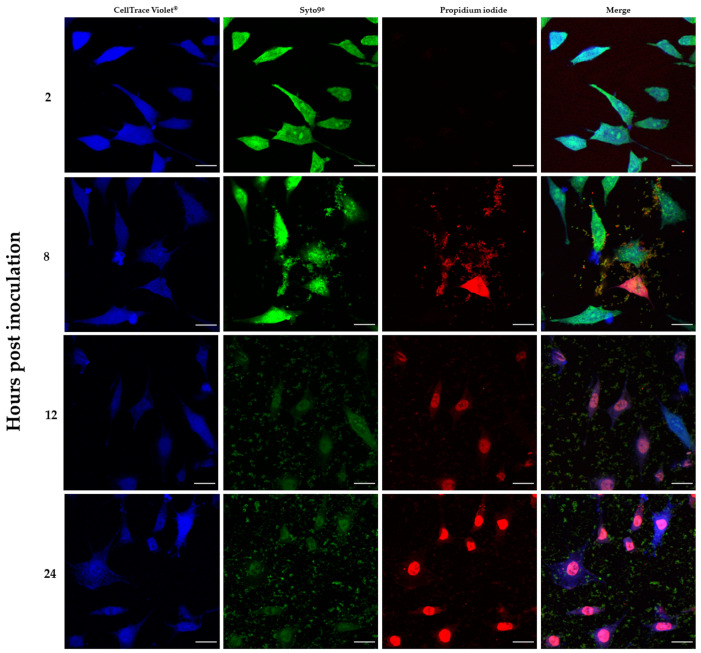
Visualization of a chronic wound infection model in vitro after 2, 8, 12 and 24 h post contamination. Live/dead staining based in Syto 9^®^ and propidium iodine combined with CellTrace Violet^®^ dye. Green—live cells, red/violet—dead cells, and blue—live eukaryotic cells. Bigger, oval/elongated shapes—fibroblasts; smaller, oval shapes—staphylococcal cells or staphylococcal cell clusters. This set of images illustrates the negative changes in viability of fibroblast cells (increasing signal from propidium iodine dye) during the co-culture process, along with undisturbed path of growth of the pathogen (increasing signal of Syto 9^®^ dye). Scale bar—20 µm.

In addition, the results provided by confocal microscopy imaging (Figure 6) allowed to additionally confirm the co-current drop of fibroblast viability and multiplication of bacterial biofilm.

The application of the above-presented various techniques (microbiological culturing, viability assays, electron and confocal microscopy) was necessary to indicate the moment of critical colonization in our in vitro model of staphylococcal biofilm-based wound infection. Knowing this specific time-point, we collected supernatant (in a real wound it would be the exudate) and performed analyses of the metabolites contained within using ^1^H-NMR spectroscopy.

The basic control setting applied in this part of the investigation was a sterile medium; the additional control settings were supernatant collected from the culture of fibroblasts alone and supernatant collected from *S. aureus* biofilm cultured alone.

**Figure 7:**
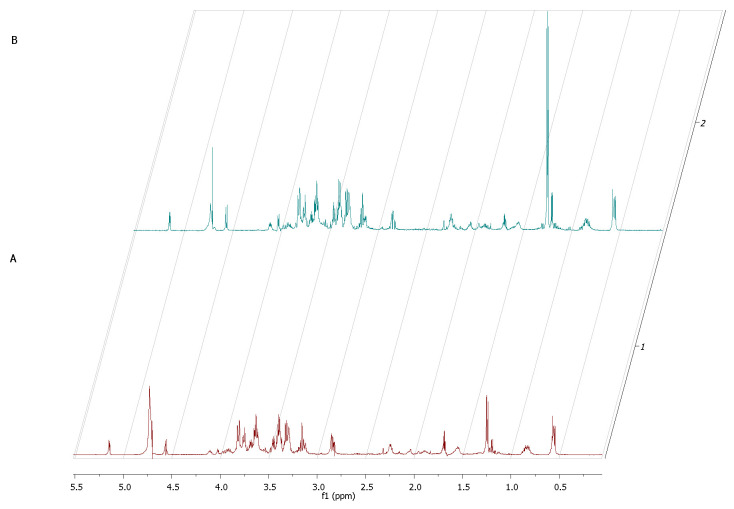
A part of ^1^H NMR spectrum of L929 fibroblasts samples after 2 h (**A**) and after 24 h (**B**).

Interestingly, the major component whose concentration dropped within the first 12 h of incubation of fibroblasts alone was glucose (σ = 5.2; 4.6; 3.9; 3.8; 3.7; 3.5; 3.4; 3.2). As this monosaccharide is the most basic source of energy, it confirms the metabolic viability of the obtained eukaryotic cell culture (Figure 7).

**Figure 8:**
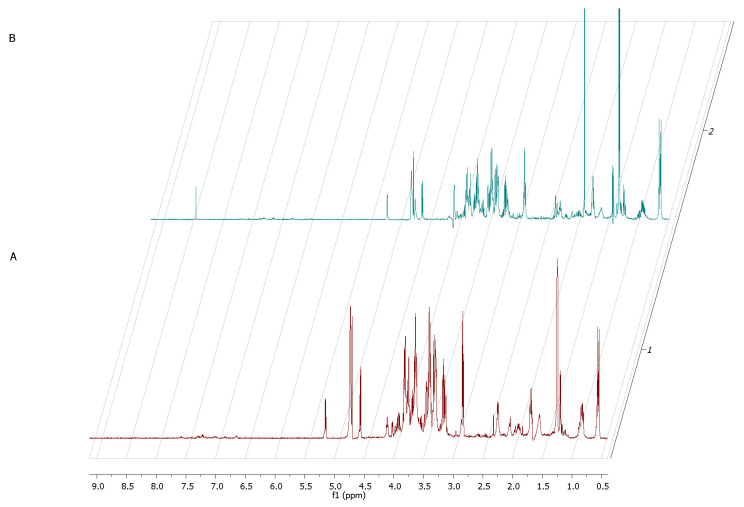
A part of the ^1^H NMR spectrum of *S. aureus* adhered cells—2 h (**A**) transforming into biofilm within 12 h (**B**).

The ^1^H NMR spectrum of staphylococcal biofilm cultured alone revealed also significant shifts in footprint metabolites after 2 and 12 h of culturing (Figure 8).

The most significant metabolites analyzed, whose content increased during the transformation from the stage of adhering (2 h of culturing) to the stage of biofilm maturation (12 h) were 3-hydroxy-3-methylglutarate and pyruvate (σ = 2.47 and 1.36, respectively).

**Figure 9:**
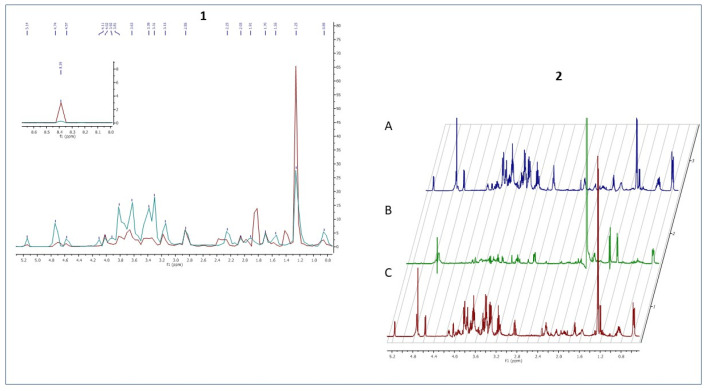
**1.** A part of the ^1^H NMR spectrum of *S. aureus* 12-h biofilm (red color) compared with 12 h co-culture of Figure 2. **2.** A part of the ^1^H NMR spectrum presenting metabolites released from L929 fibroblasts sample (**A**) or L 929 fibroblasts with *S. aureus* biofilm (**B**) or compounds recorded in pure medium sample (**C**) after 12 h of incubation.

The metabolic data presented in Figure 9 reflect this specific moment in which staphylococcal biofilm starts to devour eukaryotic fibroblast cells.

**Figure 10:**
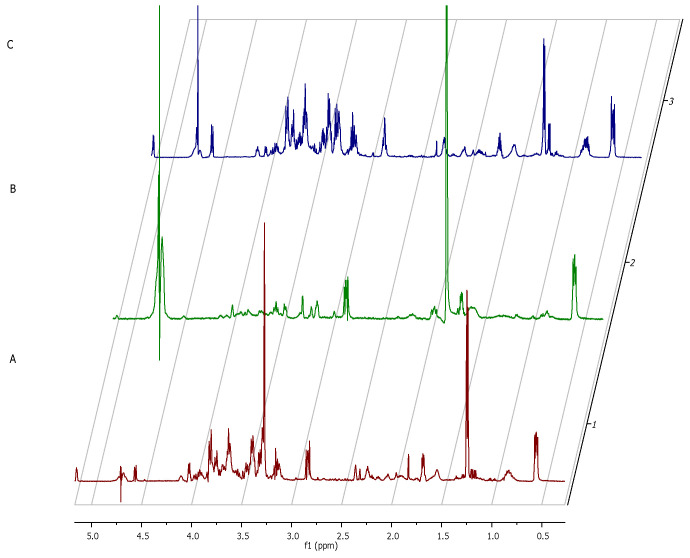
A part of the ^1^H NMR spectrum presenting metabolites released from L929 fibroblasts sample (**A**) or L929 fibroblasts with *S. aureus* biofilm (**B**) or compounds recorded from pure medium sample (**C**) after 24 h of incubation.

The only two footprint metabolites which occurred in the fibroblast-staphylococcal biofilm co-culture, but did not occur in fibrolasts cultured alone were acetate (σ = 1.82) and lactate (σ = 1.36). Finally, we analyzed also the metabolic differences in co-culture and fibroblast alone after 24h (Figure 10). The rationale behind this agenda was to catch the direction in which the changes in the metabolic profiles would develop after destruction of a majority of eukaryotic fibroblast cells.

Noteworthy, the metabolites which differentiated co-culture samples from fibroblasts in a zero/one system (i.e., metabolites present in the co-culture but non-present in the fibroblast culture) were lactate (σ =1.41 ppm), acetate (σ =1.82 ppm), formate (σ =8.39) and glucose-6-posphate (σ =4.74 ppm).

## 3. Discussion

The rise of specific disease entities is one of the consequences of the development of the so-called western civilization and lifestyle. Chronic wounds, which are particularly evident examples of such diseases, are an enormous challenge for medical care because they frequently occur secondary to such other diseases as diabetes, cardiovascular diseases, obesity or cancers [15,16,17]. Therefore, the number of reported chronic wounds has been growing annually [18].

A chronic wound is defined as a wound that fails to proceed through the normal phases of healing in an orderly and timely manner [1]. The healing process itself can be delayed by such conditions as the patient’s age, stress, medication intake or nutrition [19,20]. However, one of the crucial factors disturbing the proper healing process is the critical colonization and subsequent infection by biofilm-forming pathogens [21]. An infection within a wound may result in limb amputation or sepsis, which can lead to death if an appropriate therapy is not administered in due time.

*Staphylococcus aureus* is considered the prevailing pathogen of chronic wound infections [22]. Unfortunately, this bacteria has developed or acquired a wide range of antibiotic resistance patterns which is one of the reasons behind the more and more widespread morbidity and mortality of infected patients [23]. Currently, *S. aureus* has been classified as a multidrug-resistant microorganism (MDR) and the fatality rate for infections associated with this pathogen continues to rise [24]. Taking into consideration the specificity of biofilm development and the serious consequences of belated implementation of an appropriate wound treatment, a rapid identification (even before the first symptoms of infection occur) of the etiological factor is crucial. The classic diagnostic methods involving basic microbiological techniques like wound swabbing or biopsy-derived culturing may be biased with false-negative results due to numerous factors, of which non-homogenous spatial distribution of biofilm within the wound is one of the most important [25]. Thus, several new approaches have been developed to overcome the aforementioned issues. One of them is referred to as the Wound Check^TM^. It is based on the detection of bacterial protease activity in a chronic wound as indicative of the presence of bacterial virulence [26,27]. Noteworthy, by means of this test, one is able to detect in an indirect manner the presence of bacteria but not of biofilm. A different approach was developed by the creators of MolecuLight i:*X*^TM^—a device using fluorescence imaging to detect pathogens within the wound bed. MolecuLight i:*X*^TM^ uses the 405 nm violet-colored excitation light and, owing to optical filters, only the signal from wavelengths associated with pathogen fluorescence and tissue autofluorescence can pass through them and form a real-time image. However, although MolecuLight i:*X*^TM^ can help detect specific areas covered with biofilm [28,29], the interpretation of the obtained image is not unambiguous. Bearing the above in mind, we went about identifying biofilm-forming pathogen species by detecting their metabolites. Knowing the limitations of the current microbiological methods, we have made an attempt to expand the new diagnostic strategies. Due to the fact that we have obtained a huge amount of metabolomic data in our previous clinical study, in the present in vitro research we purposely simplified our model and we focused on three core elements: pathogen—*S. aureus*, skin cells—fibroblasts (L929 cell line), and their extracellular metabolites.

During the first stage of the investigation, we conducted an analysis of the staphylococcal biofilm development process, which allowed us to distinguish four specific phases of its formation, i.e., adhesion (Figure 2A) which occurred during the first hour post-inoculation, cell aggregation and accumulation of biomass (Figure 2B) (observed between the second and sixth hour of inoculation), and maturation (Figure 2C) (lasting to the 12th h from inoculation), based on the obtained growth curve (Figure 1). We confirmed these data using scanning electron microscopy visualizing the initial phases of biofilm development as well as the mature biofilm. Our data are consistent with the reports of other research groups [30,31,32]. Then, to reflect wound bed conditions, we have cultured the fibroblast cells until an almost confluent monolayer was obtained. In accordance with our assumption, we achieved it 16 h after seeding [33]. Next, based on the above-presented data we developed the in vitro model of chronic wound colonization/infection to examine the interaction between fibroblast and the biofilm-forming pathogen. Many attempts have already been made to create an in vitro model of chronic wounds, but according to our best knowledge, none of them succeeded in ideally mimicking the wound bed conditions. One of the currently used strategies in tissue engineering is to try to recreate the dynamics of the wound healing process by developing an in vitro model of a three-dimensional wound environment consisting of an extracellular matrix, fibroblast and keratinocyte cells, and growth factors [34,35]. During the development of our model, we tried to balance the impact of a number of factors (necessary to reflect the conditions in the wound) on the amount of generated data. We decided to analyze the dynamic interaction between two components: the microbe (*S. aureus* biofilm) and the host cells (fibroblasts). Our approach allowed us to simplify the model, to apply reliable methods and to characterize biofilm development and its effect on fibroblast’s viability. 

We analyzed the influence of staphylococcal biofilm development on the viability of fibroblast cells using several various techniques, obtaining high repeatability of outcomes. Our approach has led us to identify the specific time point (12 h post contamination) at which the process (reflecting critical colonization) occurred, and the physiological state of fibroblasts was deteriorating during co-culture with *S. aureus* biofilm. The results obtained by confocal microscopy supported the observation that the viability of the eukaryotic cells decreased during co-culture starting from 8 h of co-culture. Nevertheless, further studies are required to directly assess the pathway of fibroblast cell death as a result of necrosis/apoptosis pathways [36,37]. 

Based on these findings, we examined extracellular metabolites using ^1^H-NMR spectroscopy. First, we analyzed the control setting—supernatants collected from separately cultured fibroblasts and *S. aureus* biofilm. The main component, whose concentration dropped significantly during the 24 h of fibroblast culturing alone, was glucose (Figure 7). This finding appears to be characteristic for normal growth and high viability of fibroblast culture because glucose is the primary source of energy for most types of cells [38]. It also supports our result from the first stage of this study, that the host cell of our model was growing in a proper and undisturbed manner until contamination. Our analysis of *S. aureus* metabolites during the transformation from the initial stage of adhesion to the stage of biofilm maturation revealed that the metabolites whose levels increased most significantly were 3-hydroxy-3-methylglutarate and pyruvate. Pyruvate is the end-product of the glycolysis pathway, in which two molecules of pyruvate are generated from one glucose molecule [39]. A recent report showed that pyruvate formation that occurs in anaerobic conditions favors the staphylococcal biofilm development process [40]. According to the literature, anaerobic conditions caused by increasing cell density during biofilm growth and elevated level of pyruvate are leading to up-regulation of the biosynthetic pathways associated with biofilm formation [41]. It is also confirmed by the accumulation of glycolytic enzymes during growth under anaerobic conditions [42]. Interestingly, there were only two extracellular metabolites that have occurred in the co-culture of fibroblast and staphylococcus but did not occur in fibroblast culture at the crucial 12h of co-culture. One of them was lactate, generated from pyruvate under anaerobic growth conditions [43]. During this process, molecules of NAD^+^ are recovered. The most recent reports highlight that lactate production can influence the immunological response of the host and promote infection persistence [44]. The second identified metabolite was acetate. Some of the *S. aureus* biofilm cells from the top layers of biofilm being under aerobic conditions can excrete acetate. In turn, the cells from the bottom layers of biofilm structures switch into fermentation and produce lactate, as mentioned above. The elevated level of acetate may be the cause of inhibition of fibroblasts proliferation and may induce their apoptosis. The negative influence of acetate on the viability of mammalian cells was shown in other studies [45].

As mentioned before, there were some attempts to analyze *S. aureus* infection using in vitro models. One of the analyses was also targeted on metabolomic analysis based on the cystic fibrosis model and polymicrobial infection (*S. aureus* and *P. aeruginosa*). In this study, *S. aureus* also shifted from aerobic respiration and production of acetate to fermentation which is indicated by high accumulation of lactate probably due to oxygen deprivation [46].

In our study, after 24 h of the co-culture, most of the fibroblast cells were dead, and the majority of metabolites was secreted by staphylococcal cells. We distinguished three metabolites, namely lactate, acetate, and formate, as all of them are produced during the fermentation process. According to the literature, formate accumulation in anaerobic conditions can be caused by a rich medium [47]. One of the identified metabolites—glucose-6-phosphate—could be released from disrupted fibroblast cells. However, it could also originate from bacterial cells that have been disrupted by autolysis, which is typical for cells in matured biofilm [48].

We are aware that the main disadvantage of our study is its preliminary character: the fact that only one staphylococcal strain was tested and we are conscious of high intra-species differences within *Staphylococcus aureus*, which may affect to some extent also metabolic profiles taking place in this opportunistic pathogen. On the other hand, the metabolites we identified as potential biomarkers of the infection process are produced during anaerobic respiration processes, which are common for all staphylococci. Moreover, one should bear in mind that detection of these metabolites is easy to perform by a broad range of spectroscopic methods. The relatively simple in vitro setting we developed to mimic the interactions between fibroblasts and staphylococcal biofilm still required the application of several modern analytical techniques to draw proper conclusions from the observed phenomena. The conclusion that increased secretion of lactate, acetate and formate is correlated with robust staphylococcal biofilm development and destruction of fibroblast cells cannot be at this moment translated into any direct clinical diagnostics process. Still, we hope that preliminary data presented in this work could bring scientific environment closer to developing a new diagnostic tool aiming to rapidly detect biofilm-based critical colonization/infection in wounds and to helping patients suffering from this devastating disease.

## 4. Materials and Methods

### 4.1. Cell Lines, Strains and Culture

Fibroblast L929 cell line and reference *Staphylococcus aureus* 6538 strain from American Type Culture Collection was used for all the experiments.

The L929 mice fibroblast cells were grown using RPMI 1640 medium (Thermo Fisher Scientific, Waltham, MA, USA) supplemented with 10% fetal bovine serum (Biowest, Nuaillé, France) without any antibiotics and cultured in a humidified incubator at 37 °C and 5% of CO_2_. The cells were sub-cultivated twice a week. Prior to all experiments, the cells were seeded in standard six-well cell culture plates (Nest Biotechnology Co., Wuxi, China) at a density of 5 × 10^5^ cells per well.

The *S. aureus* ATCC 6538 strain was cultured in Brain Heart Infusion broth (Biomaxima, Lublin, Poland) medium and Columbia agar stable medium (Graso, Starogard Gdański, Poland) at 37 °C. For experiments, an overnight culture was used. Bacterial densities were estimated using a densitometer—DENSILAMETER II (ERBA, Lachema, Czech Republic) to 0.5 McFarland scale, which corresponds to approximately 1.5 × 10^8^ CFU/mL and then diluted in a 0.9% saline solution. The cells were seeded in six-well cell culture plates at a density 1.5 × 10^3^ CFU/mL for the experiment. The culture was carried out in RPMI medium supplemented with 10% FBS for 24 h.

### 4.2. Co-Culture of Fibroblast L929 Cell Line and S. aureus ATCC 6538

The co-cultures experiments were carried in conditions adapted to the eukaryotic cells described above. Briefly, fibroblast cells were seeded 5 × 10^5^ cells per well in 6-well plates and cultured in a humidified incubator at 37 °C and 5% of CO_2_ for 16 h. Then, the cells were observed under an inverted microscope (DMIL LED Leica, Germany) to check confluences. Then, the monolayer of the cells was infected with the pathogen—*S. aureus* ATCC 6538 at a density of 1.5 × 10^3^ CFU/mL. The experiment was conducted for 2, 6, 8, 16, 20 and 24 h.

### 4.3. Viability Assessment of the Bacterial and Eukaryotic Cells

#### 4.3.1. Quantitative Cultures

After each time of incubation of *S. aureus* biofilm culture and co-culture with fibroblast cells, biofilm with medium from the bottom of the wells was collected to tubes and vigorously shaken on vortex mixing for 3 min to release single cells. The Miles and Misra method was applied to determine the number of colony-forming units. The obtained bacterial suspensions were diluted 10^1^–10^7^ times. TSA (Trypticase Soy Agar, Biomaxima, Lublin, Poland) plates were divided into eight equal sectors and labeled with the dilution factor. Then, ten µL in three repeats of each dilution was dropped onto the appropriate sector of the agar surface. The plates were incubated at 37 °C for 24 h. After incubation, the microbial colonies were counted and the number of cells forming a biofilm on polystyrene and monolayer of fibroblasts was calculated.

#### 4.3.2. Cell Viability Testing with Trypan Blue Exclusion Method

The cells were detached from the wells using TrypLE™ Express (Thermo Fisher Scientific, Waltham, MA, USA) and collected to Eppendorf tubes with 2 mL of PBS (Thermo Fisher Scientific, Waltham, MA, USA). After 20 µL of the cell suspension was placed in an Eppendorf tube, 0.4% trypan blue dye (Sigma-Aldrich, St. Louis, MO, USA) was added to the cell suspension to obtain 2 dilutions. The suspension was mixed by pipetting up and down. The cells were counted using Burker Haemocytometer Counting Chamber using a light inverted microscope (Leica DMIL LED Leica, Wetzlar, Germany).

### 4.4. Visualization with Scanning Electron Microscopy and Confocal Microscopy

L929 fibroblast monolayer, *S. aureus* biofilm, and co-culture of these cell types at the chosen stage of culture were fixed in 2.5% glutarate aldehyde (Carl Roth, Karlsruhe, Germany) for 24 h at 4 °C. Then, the samples were rinsed three times with a cacodyl buffer to remove any residual of the fixative agent. The dehydration process was conducted with increasing concentrations of ethanol (30, 60, 80, 90, and 100%). The obtained samples were dried at room temperature for 15 min and coated with a 15 nm layer of carbon using a high vacuum carbon coater (ACE 600, Leica, Germany) and imaged with the ZEISS Auriga 60 scanning electron microscope (Zeiss, Germany).

The L929 cell suspension was loaded with CellTrace™ Violet (Thermo Fisher Scientific, Waltham, MA, USA), according to the protocol provided by the manufacturer. The labeled L929 cells were seeded onto glass covers in 6- well plates, 5 × 10^5^ cells per well, and incubated in a humidified incubator at 37 °C and 5% of CO_2_ for 16 h. Then, the *S. aureus* suspension at the final cell density of 2.5 × 10^3^ CFU/mL was introduced to the fibroblast monolayer. The co-cultures were incubated for 2, 8, 16 and 24 h. At time indicated, the co-culture was fixed and stained with the mixture of SYTO-9 and propidium iodide (PI) dyes with LIVE BacLight™ Bacterial Gram Stain Kit (Thermofisher Scientfic, Waltham, MA, USA) to visualize live and dead cells, respectively. The samples were imaged using an upright confocal microscope Leica SP8. Stacks of confocal 12-bit images with a voxel size of 0.117 × 0.117 × 0.999 μm were acquired using a dry 20x objective (HC PL APO CS2 20x/0.75 DRY). Cell Trace Violet, SYTO-9 and PI were excited with laser 405 nm, 488 nm and 552 nm, respectively. The emission of each dyes was collected in the range 413–465 nm, 492–534 nm and 588–631 nm, respectively for Cell Trace Violet, SYTO-9 and PI. The acquisition was performed in sequential mode. ImageJ (FiJi) was used.

### 4.5. Metabolite Extraction

After each stage of an individual culture of fibroblast cells, *S. aureus* cells, and co-culture of those cells, the media from cultures were collected. Then, medium samples were centrifuged for 20 min at 4 °C, at 4000× *g*, or 200× *g* rpm for probes from the L929 culture. The pellets of planktonic cells were discarded and supernatants were mixed with MeOH (99.6%, POCH, Gliwice, Poland) onto Thermomixer (Eppendorf, Hamburg, Germany) at 4 °C at 700 rpm for 10 min. The next step was centrifugation for 10 min at 4 °C at 12 000× *g* rpm. Excess fluid from 1 mL of the obtained supernatants was evaporated using CentriVap Benchtop Vacuum Concentrators (Labconco, Kansas City, MO, USA).

### 4.6. NMR Measurement and Data Analysis

NMR spectra of the samples were recorded using Avance II spectrometer (Bruker GmBH, Bremen, Germany) operating at a proton frequency of 600.58 MHz. All sample spectra were recorded at 300 K by CPMG pulse sequence with water presaturation (Burker notation). For each sample, 256 following scans were collected, relaxation delay of 3.5 s; acquisition time of 2.73 s; TD of 65k; line broadening of 0.3 Hz; SW of 20.01 ppm. The spectra were manually processed—phased and baseline was corrected by Topspin 3.2 software (Bruker GmBH, Bremen, Germany) and referenced to the TSP resonance signal at *δ* = 0.000 ppm. All spectra were normalized by constant sum. The NMR measured metabolites were obtained as signal integrals of non-overlapping resonances or a cluster of partly overlapping resonances. The metabolites were identified by assignments published in the literature and using Chenomx Profiler. All acquired variables were scaled to unit standard deviation.

## Data Availability

The data presented in this study are available on request from the corresponding author as the dataset obtained is planned to be applied in subsequent, chemometric analyses.
